# Lower respiratory tract infections among newly diagnosed sleep apnea patients

**DOI:** 10.1186/s12890-023-02623-0

**Published:** 2023-09-08

**Authors:** Jaana Keto, Thijs Feuth, Miika Linna, Tarja Saaresranta

**Affiliations:** 1 Department of Oral and Maxillofacial Disease, Faculty of Medicine, University of Helsinki, Helsinki, Finland; 2 Jazz Pharmaceuticals, Copenhagen, Denmark; 3 Division of Medicine, Department of Pulmonary Diseases, Turku University Hospital and University of Turku, Turku, Finland; 4 Aalto University, Helsinki, Finland; 5 University of Eastern Finland, Kuopio, Finland

**Keywords:** Sleep apnea, Lower respiratory tract infections, Pneumonia, Influenza, Multimorbidity

## Abstract

**Background:**

Sleep apnea is associated with chronic comorbidities and acute complications. Existing data suggest that sleep apnea may predispose to an increased risk and severity of respiratory tract infections.

**Methods:**

We investigated the incidence of lower respiratory tract infections in the first and second year before and after diagnosis of sleep apnea in a Finnish nationwide, population-based, retrospective case–control study based on linking data from the national health care registers for primary and secondary care from 2015–2019. Controls were matched for age, sex, hospital district, and multimorbidity status. We furthermore analysed the independent effect of comorbidities and other patient characteristics on the risk of lower respiratory tract infections, and their recurrence.

**Results:**

Sleep apnea patients had a higher incidence of lower respiratory tract infections than their matched controls within one year before (hazard ratio 1.35, 95% confidence interval 1.16–1.57) and one year after (hazard ratio1.39, 95% confidence interval1.22–1.58) diagnosis of sleep apnea. However, we found no difference in the incidence of lower respiratory tract infections within the second year before or after diagnosis of sleep apnea in comparison with matched controls. In sleep apnea, history of lower respiratory tract infection prior to sleep apnea, multimorbidity, COPD, asthma, and age greater than 65 years increased the risk of incident and recurrent lower respiratory tract infections.

**Conclusions:**

Sleep apnea patients are at increased risk of being diagnosed with a lower respiratory tract infection within but not beyond one year before and after diagnosis of sleep apnea. Among sleep apnea patients, chronic comorbidities had a significant impact on the risk of lower respiratory tract infections and their recurrence.

## Introduction

Obstructive sleep apnea is a common entity in the adult population, with age, male sex, and obesity as its main risk factors [1]. Its major detrimental factor is intermittent hypoxemia, caused by repetitive upper airway obstruction and quantified by the apnea–hypopnea index (AHI). A community-based study estimated that 17% of 50–70-year-old men and 9% of women had an AHI of ≥ 15, which is classified as moderate-to-severe sleep disordered breathing [2]. Symptomatic sleep apnea syndrome where the patient has sought medical attention and has received a sleep apnea diagnosis is rarer, with a diagnosed prevalence of 3.7%, and one-year incidence of 0.6% recently reported from a Finnish nation-wide register study [3]. Apart from hypoxemia, sleep fragmentation is another determinant of sleep apnea, impairing sleep quality and potentially leading to sleep deprivation. Severe sleep apnea may cause excessive daytime sleepiness and is associated with a substantially increased mortality risk, mainly attributable to an increased risk of acute cardiovascular events and stroke, when left untreated [4]. Moreover, sepsis patients and Covid-19 patients with comorbid sleep apnea have a higher risk of mortality compared to patients without sleep apnea [5, 6]. The risk of some types of malignant disease may also be increased in severe sleep apnea, particularly among women [7, 8].

Furthermore, sleep apnea may pose an increased risk of respiratory infections or increase their severity. In a case–control cohort, sleep apnea was more common in patients admitted for community acquired pneumonia when compared to patients admitted for other infections [9]. Several studies indicate that sleep apnea predisposes to severe influenza [10–12]. Early in the COVID-19 pandemic, sleep apnea was also identified as a risk factor for severe COVID-19 [13, 14]. However, we are not aware of previously published data on sleep apnea and lower respiratory tract infections in general (LRTI) from an unselected nationwide sleep apnea population and their matched controls, or studies where the risk of LRTI would have been inspected as a function of time from the sleep apnea diagnosis.

In this nationwide population-based study, we investigate the incidence of lower tract respiratory infections in newly diagnosed sleep apnea patients, within the two years before and two years after diagnosis.

## Methods

### Study design

**Fig. 1:**
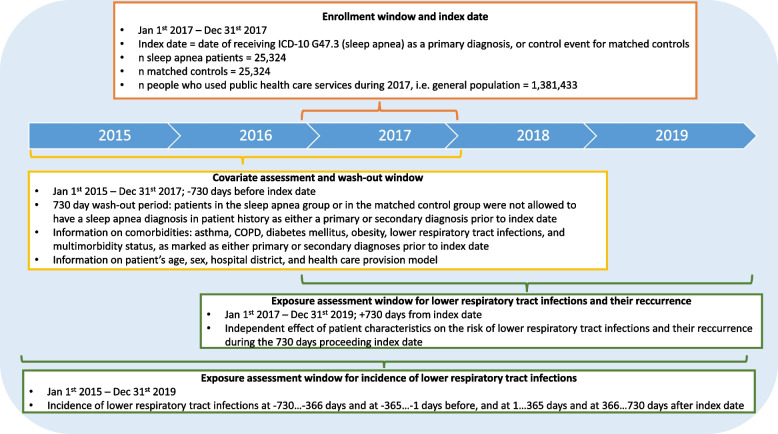
Study design

This is a population-based, retrospective observational study, based on secondary use of health care data with a complete coverage of the total population in Finland. The purpose of the study is to inspect the incidence of LRTI, and the risk of recurring LRTI among sleep apnea patients. The study design in presented in Fig. 1.

### Data sources

The study took place in Finland, a country with publicly funded universal health care, where all public health care contacts are recorded in nationwide patient registers. These comprehensive, individual-level health care data repositories, which are regularly audited for completeness and accuracy of diagnoses, are maintained by the Finnish Institute for Health and Welfare [15]. To get a holistic view of total health care use of sleep apnea patients, we included data from both primary and secondary health care, which are stored in two separate registers:

1. The Finnish Secondary Care Register (HILMO), which includes both inpatient (hospitalizations and procedures/interventions with codes) and specialised outpatient contacts (scheduled and emergency care specialist visits), and.
2. The Finnish Primary Care Register (AVOHILMO), which includes all primary health care contacts (e.g. visits to general practitioners and nurse visits) at health care centers.

The data included in these two national data repositories were linked on individual level by the Academy of Finland Research Consortium in the IMPRO research database, using the personal identity code provided for each Finnish resident [16]. For the present study, we accessed the IMPRO research database data from 2015–2019.

### Study population

#### Sleep apnea study population

Patients aged ≥ 18 years with the ICD-10 code G47.3 marked as the primary diagnosis in either primary or secondary health care in 2017 were defined as sleep apnea patients. Primary diagnosis refers to the main reason for the health care contact as documented by the physician or dentist, and secondary diagnosis refers to diagnoses that affected the diagnosis, treatment, or prognosis of the patient in the context of the current visit. The date of first sleep apnea diagnosis defined the index date for an incident cohort of 25,324 sleep apnea patients, with a wash-out period of two years. During the wash-out period, the patients were not allowed to have a G47.3 diagnosis code marked as either a primary or a secondary diagnosis in the two years preceding the index date, i.e. they had not had any contact with a physician in primary or secondary care because of sleep apnea in two years. According to Finnish national treatment guidelines, sleep apnea diagnosis requires overnight sleep study—in most cases home sleep apnea test with a level three device—in addition to medical history and physical examination. Patients with moderate-to-severe sleep apnea are referred to secondary health care for confirmation of diagnosis and initiation of therapy, which is typically continuous positive airway pressure (CPAP) or an oral appliance [17]. The validity of the G47.3 coding was > 98% in a recent study carried out in a hospital district covering 30% of the Finnish national population [18].

#### Matched controls

For each sleep apnea patient, a matched control case was randomly assigned from the Finnish Patient Registers (HILMO and AVOHILMO) in a 1:1 ratio. The matching was based on age, sex, hospital district, and binary multimorbidity status as explained below. Similarly to the sleep apnea patients, their controls had used public health care services during 2017. Hospital district was used in matching to account for potential regional differences in lifestyle and access to health care.

To account for general health status and likelihood of seeing health care professionals, we assessed the multimorbidity status of each patient. Multimorbidity was defined as two or more chronic diseases marked as a primary or secondary diagnosis in the past two years. The Finnish Institute for Health and Welfare definition of multimorbidity was used; the full list of diseases included in the definition can be found in the original classification developed by an international multidisciplinary task force [19]. The classification defines chronicity of a condition by a prolonged duration and association with either residual disability or worsening quality of life or long periods of care, treatment, or rehabilitation [19]. For sleep apnea patients, multimorbidity status was assessed prior to receiving the sleep apnea diagnosis, i.e. sleep apnea patient was multimorbid if he or she already had at least two diagnosed chronic diseases prior to the sleep apnea diagnosis. For multimorbid patients, also the year of onset of multimorbidity was used in matching.

#### General population

The general population used in the analyses consists of all ≥ 18-year-old adults who used primary or secondary health care services in Finland in 2017.

### Analyses

#### Incidence of LRTI

We report the incidence of LRTI among incident sleep apnea patients, among their matched controls, and, in order to address dynamic of infectious diseases epidemiology, among the general population.

Furthermore, we investigate whether there was a difference between the patient groups in the incidence of LRTI before and after the sleep apnea diagnosis timepoint. The exposure assessment window was divided into four 365-day periods: 730 to 366 days and 365 to 1 days before, and 1 to 365 days and 366 to 730 days after index date, i.e. sleep apnea diagnosis or control event.

Incident LRTI was defined as any of the following ICD-10 diagnoses, as recorded by the treating physician during the exposure assessment window:

- Viral LRTI: J09, J10, J11 (influenza), J12 (other viral pneumonias).
- Bacterial LRTI: J13 (pneumonia due to Streptococcus pneumonia), J14 (pneumonia due to Hemophilus influenzae), J15 (other bacterial pneumonia), J16 (pneumonia due to other infectious organisms, e.g. Chlamydia pneumoniae), J18 (Pneumonia due to unspecified organism).

#### Predisposing factors for LRTI, and its recurrence

We studied the effect of patient characteristics on the risk of LRTI among the incident sleep apnea patients and their matched controls during a two-year follow up, i.e. in an exposure assessment window of 0 to 730 days from the index date, which was defined as sleep apnea diagnosis or control event. We report the results in hazard ratios, using sex, age (under or over 65 years), binary multimorbidity status, and physician-coded asthma (ICD-10 J45), chronic obstructive pulmonary disease (COPD) (ICD-10 J44), diabetes mellitus (ICD-10 E10-14), diagnosed obesity (ICD-10 E65-66), and previous LRTI (ICD-10 J09-16, J18) from the two years preceding the index date as independent explanatory variables. We also studied the effect of the health care provision model on the risk of LRTI by using two provision models of interest as explanatory variables: a model where public health care is entirely outsourced to private producers (“outsourced health care”), and a model where primary and secondary health care are integrated (“integrated health care”). We performed the same analysis for the risk of recurring LRTI, where we defined recurring LRTI as an LRTI diagnosis occurring at least 1 month after the previous LRTI diagnosis during the exposure assessment window 0 to 730 days from sleep apnea diagnosis or control event.

#### Statistical methods

Patient characteristics for the sleep apnea cohort, their matched controls, and the general population are presented as numbers (n) and percentages (%). The two-proportion z test was used to assess the statistical significance of a difference in baseline morbidity between the sleep apnea patients and their matched controls. A *p*-value of < 0.05 was considered statistically significant.

The incidence of LRTI is presented as cases per 100 patient years, with 95% confidence intervals (CI). Risk ratio (RR) accompanied by 95% CI is calculated for the incidence of LRTI between sleep apnea patients vs. their matched controls in each 365-day exposure assessment window.

We assessed the independent effect of patient characteristics on the risk of LRTI or its recurrence by estimating hazard ratios (HR) and 95% confidence intervals (CI) with the Fine-Gray competing risk model. In the analysis, death was a competing risk for the LRTI.

Stata version 15.0 or later was used for all analyses [20].

## Results

In 2017, a total of 25,324 patients with an incident sleep apnea diagnosis were identified from Finnish national patient registers and were included in this study. Of those, 16,263 (64%) were male, and the average age was 69 years. Multimorbidity was documented in 16,030 patients (63%). Controls were randomly assigned from the 3,223,399 individuals of the general population who had used public health care services in 2017, with 1:1 matching for age, sex, hospital district, and multimorbidity status (Table 1). The sleep apnea diagnosis was made in secondary health care in 19,895 cases (79%).

**Table 1:** Demographics for the incident sleep apnea patient cohort of 2017 at the time of sleep apnea diagnosis, for the matched control population, and for the general population who had used health care services that year

	Incident sleep apnea			Matched controls			General population		
	male	female	all	male	female	all	male	female	all
n	16,263	9,061	25,324	16,263	9,061	25,324	1,381,433	1,841,966	3,223,399
Age (mean)	56.3	58.8	57.2	56.3	58.8	57.2	51.4	52.6	52.1
Age 65 + (%)	4,783 (29.4)	3,130 (34.5)	7,913 (31.2)	4,740 (29.1)	3,101 (34.2)	7,841 (31.0)	432,274 (31.3)	609,576 (33.1)	1,041,850 (32.3)
Diabetes (%)	3,324 (20.4*)	1,705 (18.8*)	5,029 (19.9*)	2,507 (15.4)	1,113 (12.3)	3,620 (14.3)	135,176 (9.8)	133,941 (7.3)	269,117 (8.3)
Obese (%)	1,053 (6.5*)	1,053 (11.6*)	2,106 (8.3*)	241 (1.5)	313 (3.5)	554 (2.2)	12,308 (0.9)	27,840 (1.5)	40,148 (1.2)
COPD (%)	572 (3.5*)	293 (3.2*)	865 (3.4*)	386 (2.4)	149 (1.6)	535 (2.1)	21,140 (1.5)	14,433 (0.8)	35,573 (1.1)
Asthma (%)	967 (5.9*)	1,137 (12.5*)	2,104 (8.3*)	657 (4.0)	620 (6.8)	1,277 (5.0)	35,029 (2.5)	72,658 (3.9)	107,687 (3.3)
Multimorbid (%)	9,478 (58.3)	6,560 (72.4)	16,038 (63.3)	9,184 (56.5)	6,386 (70.5)	15,570 (61.5)	485,917 (35.2)	740,738 (40.2)	1,226,655 (38.1)
Outsourced healthcare (%)	417 (2.6)	216 (2.4)	633 (2.5)	433 (2.7)	236 (2.6)	669 (2.6)	28,066 (2.0)	34,085 (1.9)	62,151 (1.9)
Integrated health care (%)	2,808 (17.3)	1,690 (18.7)	4,498 (17.8)	2,657 (16.3)	1,616 (17.8)	4,273 (16.9)	256,279 (18.6)	332,805 (18.1)	589,084 (18.3)

* = *p*-value < 0.05 for the difference between incident sleep apnea patients and their matched controls

### Incidence of LRTI

**Fig. 2:**
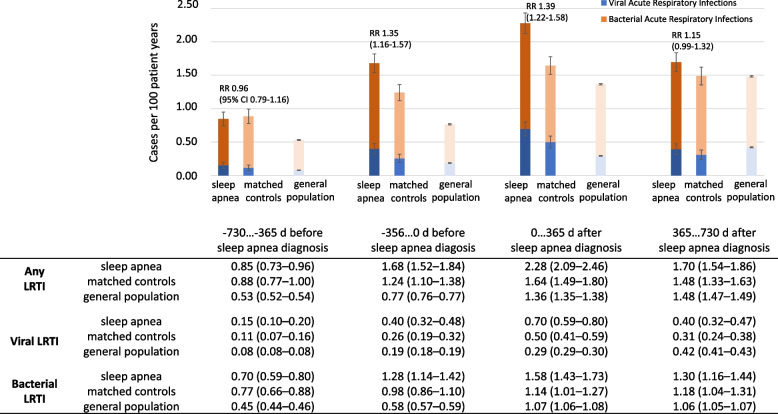
Incidence of viral and bacterial LRTI before and after diagnosis of sleep apnea or control event, stratified per year from sleep apnea diagnosis. Incidence is expressed as cases per 100 patient years, and 95% confidence intervals (CI) are depicted. Risk ratio (RR) of any LRTI with accompanying 95% CI is presented for sleep apnea patients vs. their matched controls in each time window. The background LRTI rates in the general population are depicted to account for dynamics of infectious disease epidemiology

The incidence of viral and bacterial LRTI is presented in Fig. 2, stratified by time from the sleep apnea diagnosis moment. The risk of LRTI was similar among sleep apnea patients and their matched controls more than one year before the sleep apnea diagnosis (RR 0.96, 95% CI 0.79–1.16). However, sleep apnea patients had a higher incidence of LRTI compared with their matched controls in the year preceding sleep apnea diagnosis (RR 1.35, 1.16–1.57), as well as during the year after sleep apnea diagnosis (RR 1.39, 1.22–1.58). This difference had diminished after the first year after the sleep apnea diagnosis (RR 1.15, 0.99–1.32).

### Predisposing factors for LRTI

**Fig. 3:**
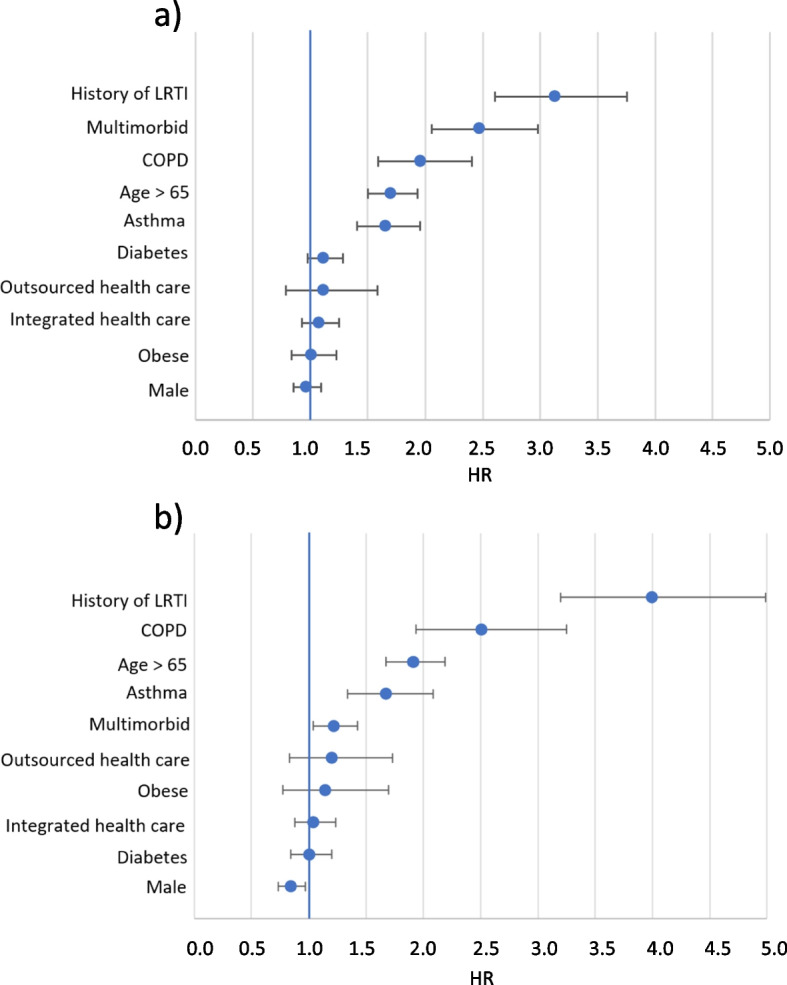
Independent effect of patient characteristics on the risk of LRTI in a two-year follow-up after sleep apnea diagnosis or control event among **a**) incident sleep apnea patients (*n* = 25,324), and **b**) their matched controls (*n* = 25,324), presented as hazard ratios (HR). The horizontal lines depict 95% confidence intervals

The independent effect of patient characteristics on the risk of LRTI in a two-year follow-up, i.e. 0…730 days after sleep apnea diagnosis, is presented in Fig. 3a for incident sleep apnea patients (*n* = 25,324). History of LRTI in the preceding two years of patient history was the strongest predictor for LRTI after sleep apnea diagnosis (HR 3.13, 95% CI 2.60–3.76), followed by multimorbidity (HR 2.47, 2.05–2.98). The comorbidity of COPD (HR 1.96, 1.59–2.40) or asthma (HR 1.66. 1.41–1.95) increased the risk for LRTI in sleep apnea patients. Older age was also associated with higher risk for LRTI (HR 1.70 for cut-off of age > 65 years old, CI 1.50–1.93). The health care provision model did not have a statistically significant effect on the risk of LRTI (HR for outsourced healthcare 1.12, 0.79–1.58; HR for integrated health care 1.08, 0.93–1.25), nor did diagnosed obesity (HR 1.01, 0.84–1.23), diabetes mellitus (1.12, 0.98–1.28), or male sex (0.97, 0.85–1.09).

Previous history of LRTI (HR 3.40, 95% CI 3.20–4.99), comorbidity of COPD (HR 2.50, 1.93–3.25) or asthma (HR 1.67, 1.34–2.09), multimorbidity (HR 1.22, 1.04–1.42), and being over 65 years old (HR 1.91, 1.67–2.19) were associated with increased risk of LRTI also among the matched controls (Fig. 3b). As in the case of sleep apnea patients, having diagnosed obesity (HR 1.14, 0.77–1.70) or diabetes mellitus (HR 1.01, 0.84–1.20) did not have a statistically significant effect on LRTI risk, and neither did the health care provision model (HR for outsourced healthcare 1.20, 0.83–1.73; HR for integrated health care 1.04, 0.88–11.24). Contrary to sleep apnea patients, male sex protected from LRTI among the matched controls (HR 0.85, 0.74–0.97).

### Predisposing factors for recurring LRTI

**Fig. 4:**
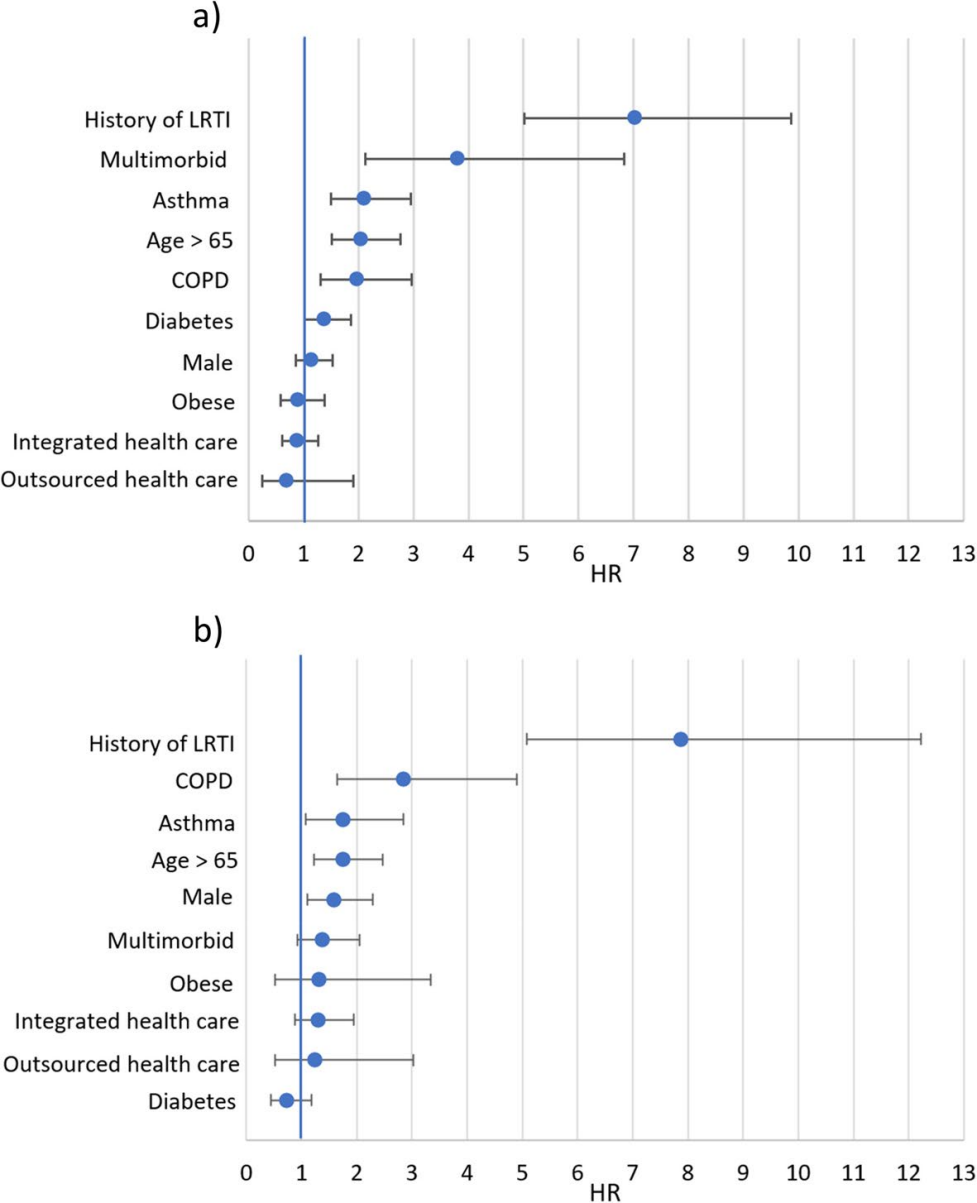
The independent effect of patient characteristics on the risk of recurring LRTI in a two-year follow-up among **a**) incident sleep apnea patients (*n* = 25,324), and **b**) their matched controls (*n* = 25,324), presented as hazard ratios (HR). The horizontal lines depict 95% confidence intervals

The independent effect of patient characteristics on the risk of recurring LRTI in a two-year follow-up from sleep apnea diagnosis is presented in Fig. 4a for incident sleep apnea patients (*n* = 25,324). History of LRTI in the preceding two years of patient history was the strongest predictor for recurring LRTI (HR 7.03, 95% CI 5.01–9.87), followed by multimorbidity (HR 3.81, 2.13–6.83. The comorbidity of asthma was associated with the greatest risk for recurring LRTI (HR 2.10, 1.50–2.95) in sleep apnea patients, followed by comorbid COPD (HR 1.97, 1.31–2.97), or diabetes mellitus (HR 1.38, 1.02–1.86). Older age (> 65 years) was also associated with higher risk for LRTI recurrence (HR 2.05, CI 1.52–2.76). The health care provision model did not have a statistically significant effect on the risk of recurring LRTI (HR for outsourced healthcare 0.69, 0.25–1.90; HR for integrated health care 0.88, 0.62–1.26). The effect of diagnosed obesity and sexwas also statistically insignificant (HR for obesity 0.90, 0.59–1.39; HR for male sex 1.15, 0.86–1.53).

The independent effects of patient characteristics for recurring LRTI were smaller for the matched controls than for the patients with sleep apnea, as depicted in Fig. 4b. The biggest independent risk factor for recurring LRTI among the matched controls was previous LRTI (HR 7.87, 95% CI 5.07–12.21), followed by COPD (2.84, 165–4.90), asthma (HR 1.75, 1.08–2.85), being over 65 years of age (HR 1.74, 1.23–2.47), and being male (HR 1.59, 1.10–2.29). Having diagnosed multimorbidity, obesity, or diabetes mellitus did not have a statistically significant effect on risk of recurring LRTI (HR 1.39, 0.92–2.05; HR 1.32, 0.52–3.35; HR 0.73, 0.45–1.17 respectively). As in the case of with sleep apnea patients, the health care provision model did not have a statistically significant effect on risk of recurring LRTI (HR for outsourced healthcare 1.25, 0.51–3.02; HR for integrated health care 1.30, 0.88–1.94).

## Discussion

In this nationwide population-based healthcare register study, we observed a markedly increased incidence of LRTI in patients with newly diagnosed sleep apnea in comparison to a control group matched for age, sex, hospital district, and multimorbidity status within one year before (RR 1.35) and after (RR 1.39) the sleep apnea diagnosis. However, this relative increase was restricted to one year before and after the diagnosis. Especially multimorbidity was a strong independent risk factor for LRTI after sleep apnea diagnosis (HR 2.47), followed by COPD, asthma, and being more than 65 years of age. The same patient characteristics also increased the risk of LRTI recurrence in sleep apnea patients: asthma, COPD, and being older than 65 years of age each roughly doubled the risk of recurring ARI, topped by having been multimorbid already prior to sleep apnea diagnosis (HR 3.81).

Several previous studies have indicated that the risk of severe respiratory infection may be increased in patients with SA. We show that this phenomenon can be observed on a nationwide population level for bacterial as well as viral infection diagnoses, but that the increased risk is only observed within one year before or after sleep apnea diagnosis.

Altered immunity might provide an explanation for increased susceptibility for severe infections. Untreated sleep apnea is associated with immune activation, as characterized by increased levels of inflammatory markers such as C-reactive protein (CRP) and proinflammatory cytokines, such as tumor necrosis factor alpha (TNF-α) and interleukin 6 (IL-6), which may be mediated by hypoxemia and related oxidative stress [21–24]. However, sleep deprivation without hypoxemia may also induce inflammation and thereby provides a second explanation for elevated markers of inflammation in untreated sleep apnea [25]. Further, sleep deprivation related to sleep fragmentation in sleep apnea seems to be associated with impaired response to immunization [26]. Chronic inflammation is associated with immune senescence and may thereby provide an explanation for increased risk of severe infection [27, 28]. Interestingly, increased levels of IL-6 were associated with risk of frequent exacerbations in asthma in a recent study, and asthma exacerbations are thought to be induced by viral infections in the majority of cases [29, 30]. Therefore, chronic inflammation induced by hypoxemia and sleep deprivation may explain the increased risk for infection in patients with untreated sleep apnea.

Sleep apnea is also associated with gastro-oesophageal reflux disease, which increases the risk of chronic aspiration and aspiration pneumonia [31]. Furthermore, the airway microbiome of patients with sleep apnea shows distinct changes in comparison to controls and may thereby predispose to respiratory infection [32].

Delay in diagnosis and treatment effect may provide one possible explanation for the time-dependent relationship between LRTI risk and SA diagnosis. In our study, the index date of sleep apnea reflects time of diagnosis rather than onset of disease. The increased risk of LRTI within one year before SA diagnosis may indicate a typical delay in diagnosis of sleep apnea when becoming relevant, as is also supported by findings by others [33]. Especially asymptomatic sleep apnea may exist already years before diagnosis, but the diagnostic delay is not reliably investigated [33, 34]. Similarly, the decrease of LRTI risk to the level of matched controls after one year after sleep apnea diagnosis may be the result of delay between diagnosis and onset of treatment, or of a delay in the preventive effect of SA treatment regarding LRTI. However, this explanation is merely hypothetical, as we had no access to data on treatment initiation for the patients included in this study. For reference, in the Turku University Hospital region, which encompasses 9% of the national population, treatment was initiated within a year in 78% of patients with a sleep apnea diagnosis in 2021– most of them with CPAP, and a minority (8%) with an oral appliance. Out of the remaining 22%, a majority received lifestyle counselling and/or positional treatment. As for compliance, some 70% of the patients on CPAP used it at least 70% of nights, for at least 4 h/night, and 20% fulfilled either the 70% or the 4 h criteria (unpublished results). According to Finnish national guidelines, treatment with a positive airway pressure device belongs to standard care of moderate and severe sleep apnea, together with lifestyle interventions [17]. As all hospitals adhere to the same national treatment guidelines, we assume, but cannot confirm, that sleep apnea treatment had been initiated in a majority of patients in our dataset consisting of newly diagnosed patients.

Alternatively, the observation of increased incidence of LRTI only within one year from sleep apnea diagnosis may be explained by a potential shorter lead time to sleep apnea diagnosis in patients presenting with LRTI, due to increased awareness and lower threshold to seek care, thereby causing bias.

Although it was beyond the scope of this study, we observed in the control group that while male sex was a protective factor for LRTI in general, it predisposed the matched controls to recurrent LRTI. A similar phenomenon was not seen among sleep apnea patients. This may be explained by differences in immunity and comorbidities predisposing to LRTI between male and female patients, as previously shown by others, or by differential health seeking behaviour between male and female patients [35, 36].

When interpreting the present results, it is worth noting that the sleep apnea patients in our data source were likely to reflect mostly symptomatic cases, as they had sought medical attention. Furthermore, sleep apnea was diagnosed in specialised secondary health care in the vast majority (79%) of cases. According to the Finnish national guidelines, only moderate to severe sleep apnea cases are primarily treated in secondary health care [17]. While the fact that all residents in Finland have access to tax-funded health care, including CPAP devices is a methodological strength of the present study, it may impact the generalisability of our results to health care systems where a sleep apnea diagnosis does not necessarily lead to treatment and subsequent follow-up.

Compared to prior studies investigating LRTI in sleep apnea patients, a major strength of the present study is the minimal selection bias and the large number of patients, as we used nationwide health care registers with full coverage of the national population. This also allowed us to match controls not only for age and sex, but also for multimorbidity status and prior comorbidities. We were also able to inspect the impact of time both before and after sleep apnea diagnosis on the risk of LRTI. Of importance, we did not focus on a single disease e.g. influenza, but included a variety of LRTIs. Furthermore, we were able to depict the epidemiological dynamics by showing incidence of LRTI in the general (non-matched) population of healthcare users in 2017.

This study has also limitations. First, despite matching, our findings may still be hampered by confounding factors that could not be accounted for, such as smoking status and other lifestyle related factors. Differently designed studies, such as selected samples with self-reported data on lifestyle and BMI, and more detailed microbiological and immunobiological LRTI data can thus complement our findings. The incidence of asthma and COPD was also slightly higher in sleep apnea patients in comparison to matched controls, possibly reflecting different comorbidity clusters of sleep apnea patients in comparison to other similarly morbid patients, such as psychiatric or cancer patients [3, 37]. Second, comorbidities may be underdiagnosed, but this accounts for the incident cases as well as for the matched controls. For instance, obesity is a common comorbidity—every third Finnish man in his sixth decade has a Body Mass Index of ≥ 30—but it was registered in only 8.3% of our patients and 2.2% of matched controls [38]. Underdiagnosis of obesity may lead to skewing towards more severe obesity in our population, as less severe cases may be more likely to remain underdiagnosed. This is a limitation of our study design, as increased weight may associate with risk of LRTI [39]. Third, mild LRTI may often remain undiagnosed by clinicians and are therefore likely to be underreported in our study. However, this effect will probably be similar in sleep apnea patients and in their matched controls. Fourth, in-depth information about type and severity of sleep apnea and its treatment is lacking, as addressed elsewhere in the discussion.

While we attempted to limit our patient population to newly diagnosed sleep apnea patients by using a clinically relevant wash-out period of two years, as well as requiring the patients to have sleep apnea as a primary diagnosis during the index year, in some cases the original sleep apnea diagnosis may date back from beyond those two years. For instance, in case a patient sought medical attention to initiate or alter their treatment again after not seeing a physician for two or more years. In an unpublished analysis of a Finnish incident sleep apnea cohort of 2019, extending the length of the wash-out period from two to three years decreased the size of the incident cohort by 8% (unpublished results). While G47.3 includes both obstructive and central sleep apnea, the proportion of central sleep apnea was considered irrelevant for the present study, as it has been found to be only 0.9% of all sleep apnea cases [40].

Overall, the biological relationship between LRTI, sleep apnea, and its treatment calls for further research with the sort of clinical data that was not available for us in the administrative health care utilisation registers this study is based on. For instance, a future prospective study could contribute to the understanding of infection risk and underlying mechanisms in sleep apnea patients by linking disease and treatment parameters to inflammatory markers.

In conclusion, in our nationwide, retrospective healthcare register-based study, the risk of LRTI was significantly increased within but not beyond the first year before and after diagnosis of sleep apnea in comparison with matched controls. The increased risk of LRTI and its recurrence was highly dependent on chronic comorbidities.

## Data Availability

The original data that support the findings of this study are available by application from the Finnish national data permit authority Findata at https://findata.fi/en/data/.

## Abbreviations

AHI: Apnea–hypopnea index
LRTI: Lower respiratory tract infections
RR: Risk ratio
HR: Hazard ratio
CI: Confidence interval
COPD: Chronic obstructive pulmonary disease
